# Some Middle School Students Want Behavior Commitment Devices (but Take-Up Does Not Affect Their Behavior)

**DOI:** 10.3389/fpsyg.2018.00206

**Published:** 2018-02-28

**Authors:** Carly D. Robinson, Gonzalo A. Pons, Angela L. Duckworth, Todd Rogers

**Affiliations:** ^1^Graduate School of Education, Harvard University, Cambridge, MA, United States; ^2^Harris School of Public Policy, University of Chicago, Chicago, IL, United States; ^3^Department of Psychology, University of Pennsylvania, Philadelphia, PA, United States; ^4^John F. Kennedy School of Government, Harvard University, Cambridge, MA, United States

**Keywords:** behavioral interventions, youth, self-control, commitment device, educational intervention, eating behavior

## Abstract

Commitment devices impose costs on one's future self for failing to follow through on one's intentions, offer no additional benefit to one's future self for following through on the intention, and people voluntarily enroll in them. Enrollment in commitment devices reflects self-awareness that one may lack sufficient self-control to fulfill one's intentions. There is little experimental research on whether school-age children possess the self-awareness necessary to enroll in a commitment device, despite evidence that children and young adolescents have many positive intentions that they fail to live up to, such as demonstrating improved school conduct or eating healthier. We report the first field experiment examining the demand for, and impact of, commitment devices among middle school students. We offered students a commitment device that imposed future costs for failing to improve in-school conduct. When presented with the opportunity to actively opt-in (default not enrolled), over one-third of students elected to enroll. When presented with the opportunity to actively opt-out (default enrolled), more than half elected to remain enrolled, showing that changing default options can increase commitment device enrollment. Despite demand for the self-control strategy, taking-up the commitment device did not affect student behavior. These findings have implications for youth-based behavioral interventions broadly, as well as those focused on eating behaviors.

## Introduction

When our best laid plans—like sticking to a healthy diet or completing an assignment on-time—go awry, we often must look inward to understand what went wrong. Time and again, despite having the necessary motivation, skills, and knowledge to act in ways that align with their long-term goals (e.g., avoid sugary foods, start the essay early), people succumb to immediate temptations that are at odds with those future aims (e.g., eating a donut, binge watching a TV series) (Milkman et al., 2008; Duckworth et al., 2014). The prevalence of self-control failures has resulted in an array of strategies that help people delay gratification and control their impulses in service of achieving their long-term goals. In the past couple decades, behavioral scientists have developed and tested strategies—often referred to as “nudges”—that encourage people to engage in desirable behaviors without restricting choice (Thaler, 2008; Benartzi et al., 2017). In this paper, we focus on one behavioral intervention that can help people accomplish their goals by anticipating and planning for future self-control failures: commitment devices (for review see Bryan et al., 2010; Rogers et al., 2014).

Commitment devices (CDs) deliberately limit future choices by allowing people to voluntarily impose costly restrictions or penalties on themselves for failing to accomplish their goals (Bryan et al., 2010; Rogers et al., 2014). For example, those who want to eat healthier might agree to deposit money into an account that they can only access again if they improve their diets, or students who want to meet an assignment deadline may ask a friend to change their Netflix password until they turn in the essay. These examples highlight two important features of CDs. First, CDs impose consequences when people fail to achieve their stated goals (e.g., losing money, locked out of Netflix account). Second, people voluntarily elect to use CDs (Rogers et al., 2014). Thus, the take-up of CDs requires individuals to possess the capacities for metacognition (i.e., awareness that their future preferences may not be aligned with their current goals) and prospection (i.e., identifying what consequence would be costly enough to make them forego immediate rewards) (Duckworth et al., 2014).

Previous experiments have shown that there can be demand among adults for CDs across various domains, including improving healthy food behaviors and academic task performance, although CD take-up rates can be very low (Rogers et al., 2014). No matter the domain, CDs require that individuals be sophisticated enough to recognize their self-control may fail without external consequences. Schwartz et al. (2014) conducted an experiment in a large national grocery chain that gave households a 25% discount on healthy foods through a rewards program. Households in the treatment condition had the opportunity to pre-commit to increasing their healthy grocery purchases by 5%, or forfeit the entire 25% discount if they failed to reach their goal. Despite no additional incentive or bonus, the study found that there was significant demand for a CD aiming to improve nutrition habits among adults: over one-third of households voluntarily agreed to increase their healthy grocery purchases or lose their 25% discount. Notably, those households that did enroll in the CD purchased significantly more health food items in the subsequent months.

CDs can be utilized to improve eating behaviors in restaurant or cafeteria settings, as well. A study found that up to one-third of customers at a fast food restaurant accepted offers from servers to cut the portions of their high calorie side dishes in half, even if they received no discount (Schwartz et al., 2012). What's more, the offer to downsize portions was more effective than the more common practice of calorie labeling (i.e., providing consumers with nutritional information) at reducing the number of calories consumed.

Another study found that a majority of college-age adults were willing to self-impose deadlines to overcome procrastination, even when those deadlines were binding and costly (i.e., each day the assignment was turned in after the self-imposed deadline, students received a 1% penalty on their overall grade; Ariely and Wertenbroch, 2002). These students were willing to risk a lower grade to apply the self-control mechanism of pre-commitment which, in turn, led to improved average grades. These studies suggest that at least a subset of adults possess the requisite skills for metacognition and prospection to recognize that CDs can help activate their self-control.

In general, people's capacities for metacognition and prospection generally improve as they age (Cunningham et al., 2007; Steinberg et al., 2009; Dimmitt and McCormick, 2012; Duckworth and Steinberg, 2015), which raises the question of whether there is demand for CDs among school-age children. For instance, in the seminal delay of gratification task where children who wait long enough get to eat two marshmallows rather than just one, preschool children tend to employ ineffective self-control strategies (e.g., imagining eating the marshmallow; Mischel and Mischel, 1987). By first grade, most children demonstrate awareness of more effective approaches for resisting the temptation to eat the sweet (e.g., covering the marshmallow). But it is not until children reach middle school that they consistently understand how and why to create a more favorable environment for delaying gratification (Mischel and Mischel, 1987).

Both in and out of school, children are constantly asked to control their impulses, delay gratification, and regulate their emotional responses (Moffitt et al., 2011). In the past few decades, the youth obesity crisis has given rise to a focus on getting children and adolescents to eat healthier, leading to a greater emphasis on how interventions can be used to beneficially modify youths' eating behaviors (e.g., Gortmaker et al., 1999; Fila and Smith, 2006). That said, effective strategies for improving eating behaviors and preventing weight gain in school-age children are in short supply (Brownell et al., 2009). Researchers have found that, while children are informed about healthy eating practices and recommendations, they find it difficult to follow-through on eating healthfully (Story and Resnick, 1986; Croll et al., 2002) even when they intend to do so (Fila and Smith, 2006). Whether the desired goal be eating healthier or turning in assignments on time, all self-control strategies depend on higher order mental processes (i.e., metacognition and prospection) to make predictions about and intervene upon lower order processes (Duckworth et al., 2014). Therefore, the extent to which children are sophisticated about their self-control problems has implications for the types of interventions that can be used to encourage desirable behaviors in youth across numerous domains (O'Donoghue and Rabin, 2001).

Even when demand for a CD exists, people might not opt-in to using it because inaction is an easier alternative (Samuelson and Zeckhauser, 1988; Kahneman et al., 1991). CDs can only change the behavior of those who agree to use them, so a central challenge is increasing usage. Research shows that take-up rates of CDs are traditionally very low (e.g., Giné et al., 2010; Royer et al., 2015), but also that changing defaults can dramatically change behavior and improve enrollment rates in a range of domains (e.g., Johnson and Goldstein, 2003; Carroll et al., 2009; Bergman and Rogers, 2017). Therefore, requiring people to opt out of a CD, as opposed to opting in, may increase the enrollment rate. Despite the potential for using defaults to influence student behaviors, research suggests most adults fail to understand or use defaults in circumstances where they might be beneficial (Zlatev et al., 2017). Children and young adolescents may stand to benefit most from changes to default decisions because they often have less control over their circumstances relative to adults (Radnitz et al., 2013).

If there is demand for CDs among school-age children, the next question is whether they are viable strategies for helping youth follow-through on their intentions. That is, even if young adolescents have the metacognitive skills to recognize they may benefit from CDs, pre-commitment may still not be an effective strategy for impacting their future actions. Given that behaviors established in youth, like healthy eating and positive school conduct, lay the foundations for adult behavior (Kelder et al., 1994; Lytle et al., 2000; Moffitt et al., 2011), identifying which strategies children can proactively enact to help with self-control failures may have long-term consequences.

## The present study

To date, there is little experimental research on whether school-age children would be willing to self-impose penalties on themselves for failing to follow-through on their intentions. In this study, we conducted the first field experiment to evaluate the plausibility of offering middle school students a CD, and then assessed whether the opportunity to pre-commit to achieving a goal would improve their behavior relative to students simply acknowledging they would like to achieve the goal. First, we explored whether middle school students have the metacognitive skills to be aware of their own limited self-control, such that they would voluntarily elect to use a CD to help them accomplish a future behavioral goal. Second, building on prior research on using defaults to increase take-up rates, we tested whether defaulting students who state they want to accomplish a goal into a CD aimed at reaching that goal (with the opportunity to opt out), increased the take-up of the CD. Third, we investigated whether offering students a CD resulted in a greater percentage accomplishing their behavioral goal. Finally, we examined whether teachers can accurately predict which students are self-aware enough of their limited self-control that they would enroll in the CD.

To answer these questions, we partnered with five middle schools in three northeastern US states that all operate under a single charter network. This charter network operates free, open-enrollment K-12 schools in under-resourced communities. All schools in the charter network utilized a behavior management system modeled after a weekly paycheck. Behavior management systems can take many forms, but tend to focus on promoting positive student behaviors (i.e., following teacher instructions, turning in assignments on-time, prosocial acts, etc.) and reducing negative student behaviors (i.e., tardiness, time-off task, fighting, etc.). Teachers and administrators use the paycheck system to track and incentivize individual students' school behaviors by awarding symbolic dollars for positive behaviors (i.e., rewards) and subtracting dollars for negative behaviors (i.e., demerits). At the end of each week, students can then spend the symbolic dollars they earn at the school store. The paycheck system provided an opportunity to study whether CDs are a useful strategy for helping children avoid self-control failures for three reasons. First, the paycheck serves as a way to quantify student behavior. Second, students presumably have a great deal of control over their school behaviors, and thus their paychecks. Finally, almost all students want to earn higher paychecks, which facilitates goal-setting.

## Materials and methods

### Participants

School enrollment at each of five middle schools ranged from 225 students to 445 students. A total of 1,632 fifth through eighth grade students were enrolled in all five schools. All 1,632 students were eligible to participate in the study, except for students excluded based on teachers' requests (i.e., students with limited English comprehension, cognitive disabilities, or individualized behavior plans that did not involve the paycheck system) (*n* = 21). Because we randomized students in the week leading up to the intervention we also sequentially excluded students after they were randomized into conditions, including students who were opted out of the study by their guardians or did not assent to participating in the study (*n* = 28), students who did not complete the intervention survey (*n* = 110), students who were absent on the day of the intervention (*n* = 143), students who could not earn a higher paycheck goal (because they had already earned the maximum goal) (*n* = 46), and students for whom the school could not provide reliable paycheck data (*n* = 79). We find no evidence that the number of students excluded differed across conditions, *p* = 0.371 (see Supplementary Materials for details). We did not collect data on student ages, but students typically begin fifth grade at age 10 or 11 and complete eighth grade at age 13 or 14. The students in the final sample (*n* = 1,205) were 52% female, and 22% of students were enrolled in fifth grade, 30% in sixth grade, 26% in seventh grade, and 22% in eighth grade. We received race and free and reduced price lunch data from four of the five schools. In these charter schools, which serve predominantly under-resourced communities, 74% of students identified as Black, 25% identified as Latino/Hispanic, and 85% received free and reduced priced lunch.

### Measures

Our main outcome measures were whether students enrolled in the CD and students' end-of-week paycheck scores. Students earn dollars toward their weekly paycheck for performing encouraged behaviors (e.g., participating in class activities, demonstrating school values). Students also can have dollars deducted from their weekly paycheck for performing discouraged behaviors (e.g., not turning in homework, not following directions). Any school faculty or staff member can award or deduct dollars from students' paychecks. At the end of each week, students receive their paycheck along with an itemized list of how and when they earned or lost dollars. Students have the opportunity to purchase items from the school store (e.g., school supplies, toys) or tickets to extracurricular activities (e.g., a pizza party) based on their paycheck balance. In four of the five schools, students start the week with $0 and there are no paycheck caps. In the last school, students start each week with $45 (i.e., a deductive payment model) and paychecks are capped at $50.

Prior to the treatment, students responded to questions about their perceptions of the paycheck. These questions were adapted from the Expectancy-Value-Cost (EVC) Scale of student motivation (Kosovich et al., 2015) to assess students' expectancy that they can earn a higher paycheck, the value students attribute to the paycheck, and the perceived costs associated with earning a good paycheck. Based on the EVC theory of motivation, expectancy reflects the extent to which a student thinks he or she can be successful in a task, value reflects the extent to which a student thinks a task is worthwhile, and cost reflects the negative aspects of engaging in a task (Wigfield and Cambria, 2010; Barron and Hulleman, 2015). We use the EVC scale to assess students' motivation for earning a good paycheck. Each item had four response options: 1 “Strongly Disagree,” 2 “Disagree,” 3 “Agree,” and 4 “Strongly Agree.” See Table 1 for the items.

**Table 1:** Items assessing students' perceptions of their paychecks and associated factors.

**No**.	**Item**	**Factor**
1	I know I can earn a better paycheck	Expectancy
2	I believe that I can be successful in earning a better paycheck	Expectancy
3	I am confident that I can earn a better paycheck	Expectancy
4	I think my paycheck is important	Value
5	I value my paycheck	Value
6	I think my paycheck is useful	Value
7	Earning a good paycheck requires too much time	Cost
8	Because of other things that I do, I don't have time to earn a good paycheck	Cost
9	I'm unable to put in the time needed to earn a good paycheck	Cost
10	I have to give up too much to earn a good paycheck	Cost

### Design and procedure

The five schools sent consent forms home to all student households. Parents and guardians had the opportunity to opt their child out of the study by returning the form to the school, or contacting a member of the research team.

In the week leading up to the intervention, the school provided the research team with students' average paycheck earnings over the past four weeks. A handful of grade leaders computed the paycheck averages based on only the prior three weeks of school (*n* = 58). The research team used the average paycheck earnings to compute a unique “paycheck goal” for each student that was 10% more than their average paycheck. For example, a student who had an average paycheck of $20 would have a paycheck goal of $22. We chose to make the paycheck goal proportional to students' average earnings as opposed to uniform (i.e., having all students' paycheck goals be the same amount) because we were concerned that low paycheck earners may become discouraged having to earn a relatively higher percentage of their average paychecks. For instance, setting a goal to earn $2 more might seem relatively achievable to students who consistently earn $40 because it is only 5% more than their average paycheck, as compared to students who consistently only earn $10 and would have to earn 20% more than their average paycheck.

We then randomly assigned students to one of three conditions: Opt-in, Opt-out, or Control. In all three conditions, students answered an initial question asking whether they wanted to set a goal to earn a paycheck of 10% over their average paycheck for the upcoming week (yes or no). In the Opt-in condition, if students answered “yes,” they were then offered a CD: They had the opportunity to pre-commit to earning their paycheck goal for that week (e.g., $22), or lose 20% of their average paycheck (e.g., $4) from the next week's paycheck if they failed to meet their goal. In the Opt-out condition, if students answered “yes” to the initial question, they were defaulted into the aforementioned CD, but could choose to opt-out of the pre-commitment by writing “I would like to drop out” at the bottom of the page. Students in the Control condition only responded to the initial question whether they wanted to set a goal to earn their paycheck goal, and were not offered the CD. Therefore, any difference between students in the Opt-in/Opt-out conditions and the Control condition can be attributed to the marginal impact of offering a commitment device relative to simply asking students whether they want to achieve a goal or not, which can be perceived as an informational nudge. Prior to the study, the research team conducted numerous pilot tests of the intervention materials with fifth graders from similar backgrounds to ensure that the directions were clear and that students understood what they were being asked to do in each condition.

In these schools, students attend homeroom at the beginning of each day, which is the classroom that students assemble in daily with the same teacher before dispersing to other classes. We performed a stratified randomization, using the students' homeroom as a stratification variable within each school. That is, to ensure that each homeroom had an equal number of students in each condition, we randomly assigned students to one of three conditions *within* their homeroom. Students were distributed across the three conditions as follows: 391 students in the Opt-in condition (32.45%), 406 students in the Opt-out condition (33.69%), and 408 students in the Control condition (33.86%).

Students completed the intervention in their homerooms via a paper-based survey. On the day of the intervention, teachers told students they had the opportunity to participate in a research study about their school experiences and paychecks. Teachers passed out pre-labeled individual envelopes to students with the help of the research team. On-site research assistants were available to answer any teacher or student questions and, when necessary, administer the survey. The envelopes ensured that student answers would not be seen by teachers and so teachers remained blind to condition assignment. The teachers read aloud the implementation script to all students, and then instructed students to open their envelopes and silently read the assent form on the first page of the survey packet.

After assenting to participating in the study, students completed the remainder of the survey. The final page of the packet varied randomly across conditions (see Supplementary Materials for sample survey). Once students completed the survey, students placed their survey packets back into the envelopes and passed the envelopes to the teacher.

The day after the survey, all students received a note from the research team in their homeroom. Students in the Control condition or who did not take-up the CD received a generic note thanking them for participating. Students in either the Opt-in or Opt-out condition who enrolled in the CD received a note reminding them of their paycheck goal for the week, and that they would lose dollars off their next week's paycheck if they failed to meet their goal.

At the end of the week, students' paychecks also included an attached note. Students in the Control condition or who did not take-up the CD again received a generic note thanking them for participating. Students who enrolled in the CD and earned their paycheck goal received a congratulatory note. Students who enrolled in the CD and failed to earn their paycheck goal were notified that they did not earn their goal, and that the deduction would be reflected in their next week's paycheck.

In the months before the study was administered to students, the research team introduced the study to teachers in staff meetings. After receiving an overview of the study, each teacher was asked to predict whether each student in their homeroom class would take-up the CD or not.

In accordance with human subject protection, this procedure and experiment were approved and overseen by the Harvard University Institutional Review Board (Protocol #13-2091). Before analyzing the data, we registered our study design, hypotheses, and analysis plan on *AEA RCT Registry*.

### Analytic details

We conducted regression analyses with paycheck earnings as the dependent variable, controlling for students' homeroom, average pre-treatment paycheck earnings, and pre-treatment math grades. For regression analyses with CD take-up as the dependent variable, we controlled for students' homeroom and pre-treatment math grades. All results are robust to the exclusion of these covariates. We do not include covariates in any other analyses. We evaluated our hypotheses using 95% confidence intervals to emphasize the range of plausible values for the treatment effect, in addition to *p*-values (Cumming, 2014).

## Results

### Balance equivalence and descriptive statistics

We checked to ensure the three conditions were balanced across covariates. For a breakdown of participating students' demographics by condition see Table 2. A multinomial logistic regression predicting condition assignment with available pre-treatment variables for all students, such as math grade, proportion of female students, proportion of students in each school, proportion of students in each grade, and average pre-treatment paycheck earnings, was not statistically significant [*LR* $\chi_{\left(20\right)}^{2}$ = 12.29, *p* = 0.906]. For the four schools that provided student race information, we checked for whether students' race was balanced across conditions. The distribution of race in the Control, Opt-in and Opt-out conditions, respectively, were 72.87, 75.43, 73.33% for Black, $\chi_{\left(2\right)}^{2}$ = 0.58, *p* = 0.749, and 24.61, 26.62, 23.81% for Hispanic/Latino, $\chi_{\left(2\right)}^{2}$ = 0.68, *p* = 0.713.

**Table 2:** Balance table and descriptive statistics.

	**Control**	**Opt-in**	**Opt-out**	**Total**	***p*-value**
Average Paycheck Pre-Treatment	$41.27	$40.47	$42.25	$41.34	0.372^a^
Female	56.37%	48.97%	51.49%	52.33%	0.103^b^
School 1	13.73%	13.81%	12.32%	13.28%	0.953^b^
School 2	23.28%	22.76%	24.38%	23.49%	
School 3	22.30%	24.81%	22.17%	23.07%	
School 4	16.18%	17.39%	17.24%	16.93%	
School 5	24.51%	21.23%	23.89%	23.24%	
Math Grade Pre-Treatment	81.42	81.18	82.04	81.55	0.566^a^
5th grade	23.04%	22.25%	21.43%	22.24%	0.995^b^
6th grade	29.41%	29.41%	30.79%	29.88%	
7th grade	25.74%	26.85%	25.37%	25.98%	
8th grade	21.81%	21.48%	22.41%	21.91%	

a *p-value from a F-statistic*.

b *p-value from a χ^2^ statistic*.

Students earned an average pre-treatment paycheck of $41.34 (*SD* = 17.91) and an average post-treatment paycheck of $39.98 (*SD* = 21.77). In the weeks leading up to the intervention, students in higher grades tend to earn higher paychecks than students in lower grades, *B* = 1.41, *SE* = 0.48, *t* = 2.93, *p* = 0.003, CI [0.47, 2.36]. On average, eighth graders earned $44.17, seventh graders earned $41.62, sixth graders earned $39.99, and fifth graders earned $40.03.

### Perceptions about the paycheck

Students' responses to the adapted EVC Scale of student motivation demonstrated how they perceived their weekly paychecks. Specifically, we were interested in how motivated students were to earn good paychecks as a check to determine if improving their paycheck was a reasonable goal for students to set. A principal component analysis with varimax rotation confirmed the latent 3-factor structure as suggested by Kosovich et al. (2015), KMO = 0.83; $\chi_{\left(45,\;1,103\right)}^{2}$ = 4123.83, *p* < 0.001; see Supplementary Materials). In favor of a more parsimonious analysis, we computed the simple average score of the items for each factor. The mean score for students' perceived expectancy of the paycheck was 3.35, *SD* = 0.51, *SE* = 0.015, *CI* [3.33, 3.38]. The mean score for students' perceived value of the paycheck was 2.82, *SD* = 0.75, *SE* = 0.022, *CI* [2.78, 2.87]. The mean score for students' perceived costs associated with earning a good paycheck was 2.06, *SD* = 0.62, *SE* = 0.018, *CI* [2.03, 2.10]. These results suggest that, on average, students agreed that they could earn a better paycheck and that the paycheck was valuable, but disagreed that the costs associated with earning a good paycheck were too high. Overall, students appear to be motivated to earn good paychecks.

### Desire to set a goal to earn a higher paycheck

Across the three conditions, 75.6% of students said that they *wanted* to set a goal to increase their paycheck by 10%. In the Control condition, which was not influenced by the CD narrative in the survey, 79.9% of students indicated they wanted to set the goal. The percentage of students that wanted to set the goal was significantly different across conditions with 80.1% of students in the Opt-in condition indicating they wanted to set the paycheck goal, but only 67% of students in the Opt-out condition expressing interest in setting the paycheck goal, $\chi_{\left(2\right)}^{2}$ = 24.59, *p* < 0.001. This difference may be due to the fact that if students in the Opt-out condition read to the bottom of the page before making their decision, they would have realized they would have to take an extra step to write, “I would like to drop out” if they did not want to take-up the CD. Students in the Opt-in condition only had to respond yes or no. We find no other evidence that students assigned to the Opt-out condition differ from students assigned to the Opt-in and Control conditions on any other dimensions.

### Take-up of commitment device

**Figure 1:**
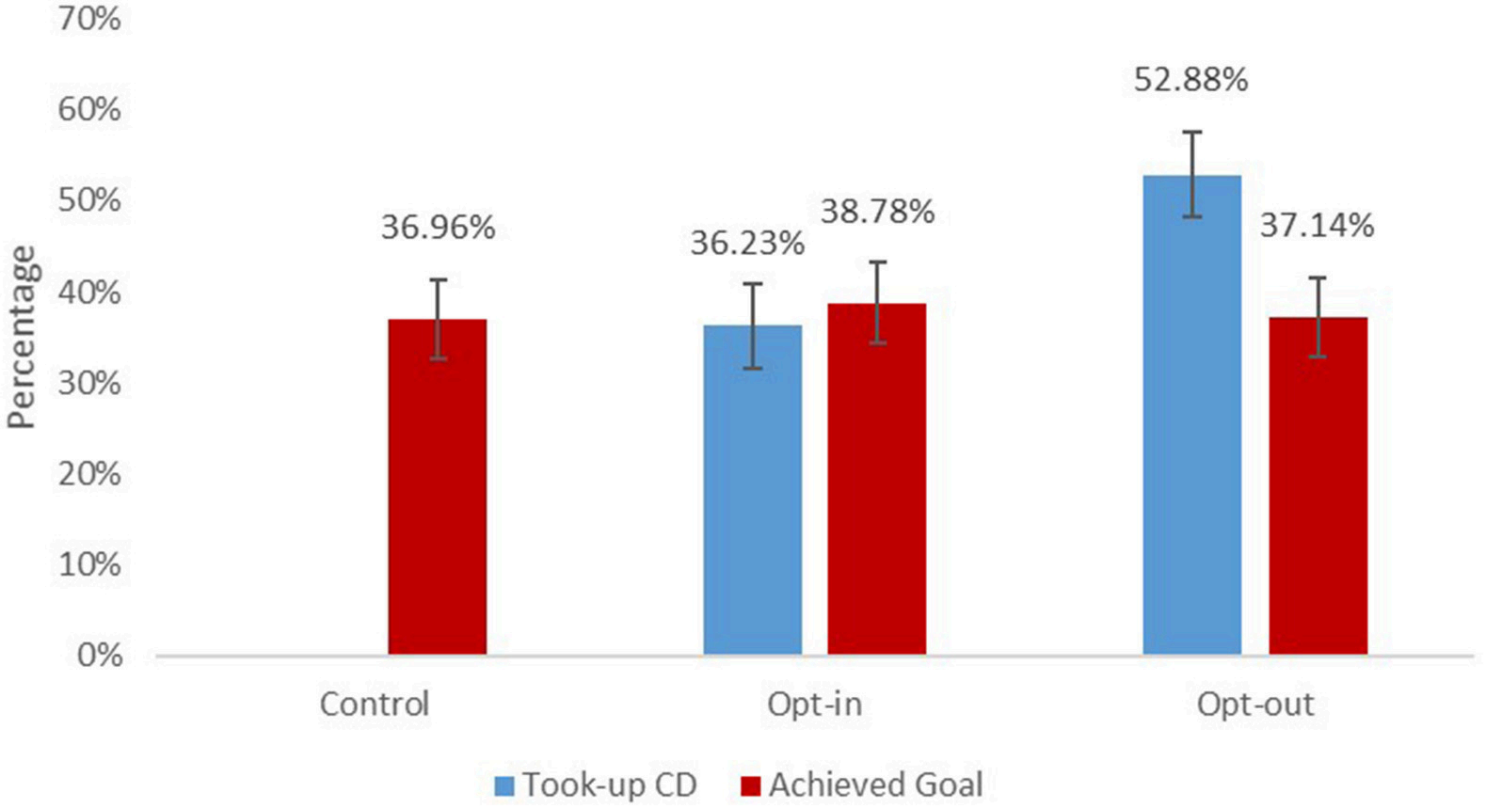
Percentage of students who took-up commitment device and achieved their goal by treatment condition. Error bars represent 95% CI. Estimates are from a logistic regression that controlled for students' homeroom and pre-treatment math grade. Students in the Control condition did not have the opportunity to take-up the commitment device.

We expected that defaulting students into enrolling in the CD would increase take-up rates. A logistic regression confirmed this hypothesis, and Figure 1 shows that a significantly higher percentage of students enrolled in the CD in the Opt-out condition (52.9%) than in the Opt-in condition (36.2%), *B* = 0.76, *SE* = 0.16, *z* = 4.79, *p* < 0.001, *CI* [0.45, 1.08]. Student age did not affect demand, as CD take-up did not differ by grade.

### Teachers' prediction of students' commitment device take-up

We expected that teachers' predictions of students' take-up of the CDs would be uncorrelated with actual take-up of the CD. For this analysis we used a logistic regression, clustering the standard errors by teacher and homeroom. When we pooled the two treatment conditions together, we found no association between teacher predictions and actual enrollment, *B* = 0.257, *SE* = 0.196, *z* = 1.31, *p* = 0.190, *CI* [−0.127, 0.641]. However, when we distinguished between treatment conditions, teachers were somewhat more accurate in their predictions when the student was assigned to the Opt-in condition. That is, a teacher's prediction that a student in the Opt-in condition would take-up the CD was associated with a 12-percentage point increase in the probability of that student enrolling in the CD, *B* = 0.535, *SE* = 0.239, *z* = 2.23, *p* = 0.026, *CI* [0.065, 1.004].

### Commitment device and student paychecks

We hypothesized that students in the treatment conditions who had the opportunity to enroll in the CD (i.e., the Opt-in and Opt-out conditions) would earn higher paychecks as compared to students in the Control condition. However, we found no evidence to support the hypothesis that the CD offer impacted students' subsequent behavior across multiple model specifications.

An intent-to-treat (ITT) analysis using OLS regression showed that there was no significant difference on end-of-week paycheck earnings between the Control condition and either of the CD conditions. Model 1 in Table 3 confirms that, compared to the Control condition, both confidence intervals for the Opt-in condition estimate and Opt-out condition estimate include zero. In both the Opt-in and Opt-out conditions the estimated coefficients are very small (0.393 and −0.096 dollars, respectively). These coefficients translate to effect sizes of 0.018 and −0.004 standard deviations, respectively, further signifying that we have no evidence that the treatment groups differed from the control group in the population. The results remain unchanged when limiting the analysis to students who initially indicated that they wanted to set the paycheck goal (see Model 2 in Table 3).

**Table 3:** Commitment device and student paycheck results.

**Outcome**	**Paycheck (1)**	**Paycheck (2)**	**Met goal (3)**	**Paycheck (4)**	**Paycheck (5)**
Opt-in	0.393 (−1.648, 2.434)	0.396 (−1.909, 2.701)	0.018 (−0.044, 0.080)		
Opt-out	−0.096 (−2.109, 1.916)	0.608 (−1.772, 2.988)	0.002 (−0.059, 0.062)		
Take-up CD × Opt-in				1.130 (−4.495, 6.754)	
Take-up CD × Opt-out				−0.189 (−3.908, 3.531)	−0.833 (−4.8, 3.135)
Analysis	ITT	ITT	ITT	TOT	TOT
Excluded		Did not want to set paycheck goal			Control group
*N*	1,193	900	1,178	1,193	788
Coefficients	$	$	Margins	$	$

^*^p < 0.1; ^**^p < 0.05; ^***^p < 0.01.

*95% Confidence Intervals are given in parenthesis. All models control for homeroom, average pre-treatment paycheck, and pre-treatment math grade. “Take-up CD” variables in model 4 and 5 are instrumented using condition assignment (CD = commitment device). The sample size reduction in model 3 is due to strata that were excluded due to perfect prediction*.

Additionally, having the opportunity to enroll in the CD did not affect the probability that students met their paycheck goal (see Model 3 in Table 3). The difference in goal achievement between conditions is not statistically significant: 37% of students in the Control condition met their paycheck goal, while 39% of students in the Opt-in condition and 37% of students in the Opt-out condition met their paycheck goal.

Finally, we performed a treatment-on-the-treated (TOT) analysis, using condition assignment as an instrument for CD take-up (a two stage least squares [2SLS] regression). We found no significant differences between the Control condition and either treatment condition (see Models 4 and 5 in Table 3). The coefficients in Model 4 represent the difference in paycheck scores between each treatment condition and the Control condition, while the coefficient in Model 5 shows the difference in paycheck scores between the Opt-in and Opt-out conditions.

Standardizing students' paychecks within each school did not meaningfully change the results nor did conducting a difference-in-difference regression on student paycheck scores (see Supplementary Materials).

### Pre-treatment paycheck performance

As an exploratory analysis, we investigated whether pre-treatment paycheck average was correlated with enrolling in the CD and meeting the paycheck goal. In both treatment conditions, students' pre-treatment paycheck average was negatively associated with taking up the CD, *B* = −0.021, *SE* = 0.004, *z* = −4.81, *p* < 0.001, *CI* [−0.029, −0.012]. In the Control condition, students' pre-treatment paycheck average was negatively associated with meeting their paycheck goal, *B* = −0.021, *SE* = 0.006, *z* = −3.28, *p* = 0.001, *CI* [−0.033, −0.008].

## Discussion

As researchers, practitioners, and policymakers increasingly turn to behavioral science, those working with school-age children must consider which nudges are developmentally appropriate for youth. Our study starts to shed light on whether CDs could be an effective strategy for helping school-age children follow-through on their intentions. While we advance knowledge about school-age children's metacognition and how defaults may impact the take-up of CDs, we do not find evidence that this CD impacts middle school students' school behavior.

### Students' metacognition

This study reveals that middle school students are more self-aware of their limited self-control than we might realize. When given the opportunity, over one-third of students in the Opt-in condition voluntarily elected to enroll in a CD to help them follow-through on their goal to improve their school conduct. While this study focuses on a more general in-school behavior, there is little reason to believe this capacity for metacognition would not transfer to other domains (e.g., nutrition and exercise). Therefore, a sizable fraction of students appear to possess the metacognitive skills to understand that there may be a gap between their current goals (e.g., to earn a higher paycheck, to eat more healthfully) and their future preferences (e.g., talking to their friend during class, selecting pizza over salad in the cafeteria).

The demand among middle school students to employ a tool that will help them follow-through on an intended behavior, even when failure to follow-through is associated with negative consequences, could inform future strategies that attempt to improve young adolescents' self-control. Instead of hoping school-age children simply develop more “willpower” when facing temptations that conflict with their long-term goals, future interventions can leverage young adolescents' willingness to proactively modify their situation in ways that reduce the desirability of succumbing to anticipated in-the-moment impulses (Duckworth et al., 2014, 2016).

### Defaults increase commitment device take-up

Despite the potential of using pre-commitment as a self-control strategy, enrollment rates of CDs tend to be low (Rogers et al., 2014). The desire to increase the percentage of people who take-up a CD is often at odds with the voluntary aspect of the approach. We found that requiring students to opt-out of the CD, as opposed to convincing them to proactively opt-in, maintained personal agency and increased the percentage of students who enrolled in the CD by 46% (or 17 percentage points). This finding builds on past research that opt-out or default framing increases take-up among adults across many domains (e.g., taxes and organ donation). We contribute to this body of evidence by demonstrating that defaults are also effective in the educational domain and with middle school-age children.

As hypothesized, teachers were not very accurate when predicting which of their students would take-up the CD. However, teachers were more accurate in predicting whether a student would take-up the CD if they had to proactively opt-in. This suggests that, like adults in other fields (Zlatev et al., 2017), teachers and other adults may not be aware of the impact that defaults can have on youth participation, and that default choice architecture holds potential for increasing youth-focused intervention enrollment rates across many domains.

### When commitment devices fail

Despite finding that there was demand for the self-control strategy, we found no evidence that enrollment in the CD impacted student behavior as measured by their paychecks. Compared to students in the control condition, students in the treatment conditions did not earn higher paychecks, nor were they more likely to meet their paycheck goals. We do not interpret our results as evidence that CDs *cannot* impact student behavior in any domain. Rather, we interpret them as suggesting that the present CD intervention was ineffective (i.e., the small effect sizes and that the confidence intervals for the treatment effect included zero). Absence of evidence, after all, is not the same as evidence of absence.

Additionally, our experiment cannot speak to why the CD did not significantly improve student behavior, but there are a few potential explanations that we hope can be addressed in future research.

First, the standard goal of increasing their average paycheck by 10% for all students meant that students with higher paycheck averages had larger and more difficult goals to achieve. For example, a student with an average paycheck of $50 needed to earn $5 more than usual, or risk losing $10. But, a student with an average paycheck of $10 only needed to earn $1 more dollar than usual, and only risked losing $2. The relatively larger goal could have decreased the motivation of students with higher average paychecks before the intervention to take-up the CD. In support of this explanation, we found that having a higher pre-treatment average paycheck was associated with lower likelihood of enrolling in the CD. Thus, it is possible that at least a subset of these students with high initial paychecks acted rationally, recognizing that the paycheck goal was unrealistic and weighing that against the relatively higher stakes. Future studies might explore how the target goal impacts the enrollment and effectiveness of CDs.

Second, while young adolescents may possess the metacognitive awareness that they have limited self-control, they may nevertheless struggle to make use of self-control strategies. Self-control tends to improve as children age (Eisenberg et al., 2014; Duckworth and Steinberg, 2015), and middle school students may be too young to effectively employ CDs. This study suggests that more research is needed to determine at what age CDs become a viable strategy for discouraging undesirable behavior.

Finally, middle school students may lack the requisite capacity for prospection which is needed to identify what consequences, specifically, will be costly enough to motivate their future selves to forego immediate temptation (Duckworth et al., 2014). That is, students may believe that the threat of losing 20% of their average paycheck would incite them to avoid temptation, but they incorrectly predicted the extent to which they valued their paycheck relative to their short-run impulses. Mochon et al. (2016) found that shoppers for whom eating behavior CDs were effective (i.e., they met their goal of purchasing more nutritional foods) were those that had the most to lose by failing to meet their pre-commitment, suggesting the consequence must be costly enough to evoke behavior change. Therefore, understanding the extent to which school-age children can predict what costs are associated with controlling their impulses will be an important next step. This will be especially important if children need to forecast what will motivate them, for example, to follow-through on their goal of eating healthy in the face of the daily onslaught of unhealthy foods they likely encounter (Harris et al., 2009; Lee, 2012).

Our findings suggest that CDs intended to help middle school students follow-through on their intentions may be more effective if they target specific, momentary behaviors, rather than ambiguous behaviors over long periods of time. For instance, youth may be better served by an intervention that emulates Schwartz et al. (2012) portion downsizing intervention where the target behavior is restricted at the moment students express demand for the CD, as opposed to offering a CD that requires students to modify their own future behaviors without additional self-control reinforcements.

### Limitations

There a few limitations to consider when interpreting the results of this study. First, because we focused on a school network for its paycheck system, our sample is necessarily limited to the student populations these schools serve. In this case, these schools serve students from underserved neighborhoods which may limit the generalizability of these results. Future research might explore how middle school students from other backgrounds react to the opportunity to take-up a CD. Second, our study required students to be in attendance on a single school day and provide their assent to participate. While there is no reason to think that our study would influence students' attendance, it is possible that students who were absent or did not assent to participate would have lower baseline levels of self-control, leaving us with a sample of students who have higher self-awareness of their self-control limitations. Third, compared to students in the Control and Opt-in conditions, students assigned to the Opt-out condition were less likely to say they wanted to earn a higher paycheck. We hypothesize that some students in the Opt-out condition may have read to the bottom of the page first and realized that indicating they wanted to earn a higher paycheck would require additional effort (writing “I would like to drop out”) to withdraw from the CD. We do not observe any other differences between the conditions, but it is possible that there are unobservable differences which would limit the conclusions we can draw from the results. Finally, because we had a limited window for conducting the study (one week) we were not able to test the impact of the intervention on students' behavior in subsequent weeks. Thus, we do not know if students who took-up the CD and lost 20% of their earnings because they failed to meet their paycheck goal would have learned from their mistakes. Future studies on CDs might explore how people learn and adapt their behaviors after self-control failures.

## Conclusion

While the importance of self-control to healthy development is well-established, there are few examples of translational research on self-control interventions targeting youth in real-world settings. The present study suggests that some adolescents are willing to impose penalties on themselves for failing to reach their goals, and that a default framing can increase the take-up of CDs. That said, although CDs have been shown to help adults exercise self-control in the short-term in service of achieving their long-term goals, we did not find evidence that CDs are effective at encouraging middle school students to improve their school behaviors. Future research should explore at what age, in what domains, and in what form nudge interventions, such as CDs, are developmentally appropriate for effectively improving self-control.

## Author contributions

The study was conceived by TR and AD. TR, CR, and AD developed the materials. TR and CR oversaw implementation of the study. GP and CR led the analysis with feedback from TR and AD. All the authors contributed to writing the manuscript.

### Conflict of interest statement

The authors declare that the research was conducted in the absence of any commercial or financial relationships that could be construed as a potential conflict of interest.

## References

[B1] Ariely D. Wertenbroch K. (2002). Procrastination, deadlines, and performance: self-control by precommitment. Psychol. Sci. 13, 219–224. 10.1111/1467-9280.00441 12009041

[B2] Barron K. E. Hulleman C. S. (2015). Expectancy-value-cost model of motivation, in International Encyclopedia of the Social and Behavioral Sciences, 2nd edn., Vol. 8, ed Wright J. D. (Oxford: Elsevier Ltd.), 503–509.

[B3] Benartzi S. Beshears J. Milkman K. L. Sunstein C. R. Thaler R. H. Shankar M.. (2017). Should governments invest more in nudging? Psychol. Sci. 28, 1041–1055. 10.1177/0956797617702501 28581899 PMC5549818

[B4] Bergman P. Rogers T. (2017). Is this technology useless? How seemingly irrelevant factors affect adoption and efficacy in HKS Faculty Research Working Paper Series, RWP17–021.

[B5] Brownell K. D. Schwartz M. B. Puhl R. M. Henderson K. E. Harris J. L. (2009). The need for bold action to prevent adolescent obesity. J. Adolesc. Health 45, S8–S17. 10.1016/j.jadohealth.2009.03.004 19699441

[B6] Bryan G. Karlan D. Nelson S. (2010). Commitment devices. Annu. Rev. Econ. 2, 671–698. 10.1146/annurev.economics.102308.124324

[B7] Carroll G. D. Choi J. J. Laibson D. Madrian B. C. Metrick A. (2009). Optimal defaults and active decisions. Q. J. Econ. 124, 1639–1674. 10.1162/qjec.2009.124.4.1639 20041043 PMC2798815

[B8] Croll J. Neumark-Sztainer D. Story M. Ireland M. (2002). Prevalence and risk and protective factors related to disordered eating behaviors among adolescents: relationship to gender and ethnicity. J. Adolesc. Health 31, 166–175. 10.1016/S1054-139X(02)00368-3 12127387

[B9] Cumming G. (2014). The new statistics: why and how. Psychol. Sci. 25, 7–29. 10.1177/0956797613504966 24220629

[B10] Cunningham W. A. Zelazo P. D. Packer D. J. Van Bavel J. J. (2007). The iterative reprocessing model: a multilevel framework for attitudes and evaluation. Soc. Cogn. 25, 736–760. 10.1521/soco.2007.25.5.736

[B11] Dimmitt C. McCormick C. B. (2012). Metacognition in education, in APA Educational Psychology Handbook, Vol. 1, Theories, Constructs, and Critical Issues, eds Harris K. R. Graham S. Urdan T. C. McCormick C. B. Sinatra G. M. Sweller J. (Washington, DC: American Psychological Association), 157–187.

[B12] Duckworth A. L. Gendler T. S. Gross J. J. (2014). Self-control in school-age children. Educ. Psychol. 49, 199–217. 10.1080/00461520.2014.926225

[B13] Duckworth A. L. Gendler T. S. Gross J. J. (2016). Situational strategies for self-control. Perspect. Psychol. Sci. 11, 35–55. 10.1177/1745691615623247 26817725 PMC4736542

[B14] Duckworth A. L. Steinberg L. (2015). Unpacking self-control. Child Dev. Perspect. 9, 32–37. 10.1111/cdep.12107 25821515 PMC4372146

[B15] Eisenberg N. Duckworth A. L. Spinrad T. L. Valiente C. (2014). Conscientiousness: origins in childhood? Dev. Psychol. 50, 1331–1349. 10.1037/a0030977 23244405 PMC3610789

[B16] Fila S. A. Smith C. (2006). Applying the theory of planned behavior to healthy eating behaviors in urban native American youth. Int. J. Behav. Nutr. Phys. Activ. 3:11. 10.1186/1479-5868-3-11 16734903 PMC1501033

[B17] Giné X. Karlan D. Zinman J. (2010). Put your money where your butt is: a commitment contract for smoking cessation. Am. Econ. J. Appl. Econ. 2, 213–235. 10.1257/app.2.4.213

[B18] Gortmaker S. L. Peterson K. Wiecha J. Sobol A. M. Dixit S. Fox M. K.. (1999). Reducing obesity via a school-based interdisciplinary intervention among youth: planet health. Arch. Pediatr. Adolesc. Med. 153, 409–418. 10.1001/archpedi.153.4.409 10201726

[B19] Harris J. L. Bargh J. A. Brownell K. D. (2009). Priming effects of television food advertising on eating behavior. Health Psychol. 28, 404–413. 10.1037/a0014399 19594263 PMC2743554

[B20] Johnson E. J. Goldstein D. (2003). Do defaults save lives? Science 302, 1338–1339. 10.1126/science.1091721 14631022

[B21] Kahneman D. Knetsch J. L. Thaler R. H. (1991). Anomalies: the endowment effect, loss aversion, and status quo bias. J. Econ. Perspect. 5, 193–206. 10.1257/jep.5.1.193

[B22] Kelder S. H. Perry C. L. Klepp K. I. Lytle L. L. (1994). Longitudinal tracking of adolescent smoking, physical activity, and food choice behaviors. Am. J. Public Health 84, 1121–1126. 10.2105/AJPH.84.7.1121 8017536 PMC1614729

[B23] Kosovich J. Hulleman C. Barron K. Getty S. (2015). Developing a practical measure of motivation: expectancy-value-cost in middle school science and mathematics. J. Early Adolesc. 35, 790–816. 10.1177/0272431614556890

[B24] Lee H. (2012). The role of local food availability in explaining obesity risk among young school-aged children. Soc. Sci. Med. 74, 1193–1203. 10.1016/j.socscimed.2011.12.036 22381683

[B25] Lytle L. A. Seifert S. Greenstein J. McGovern P. (2000). How do children's eating patterns and food choices change over time? Results from a cohort study. Am. J. Health Promot. 14, 222–228. 10.4278/0890-1171-14.4.222 10915532

[B26] Milkman K. L. Rogers T. Bazerman M. H. (2008). Harnessing our inner angels and demons: what we have learned about wantshould conflicts and how that knowledge can help us reduce short-sighted decision making. Perspect. Psychol. Sci. 3, 324–338. 10.1111/j.1745-6924.2008.00083.x 26158952

[B27] Mischel H. N. Mischel W. (1987). The development of children's knowledge of self-control strategies, in Motivation, Intention, and Volition, eds Halisch F. Kuhl J. (Berlin; Heidelberg: Springer), 321–336.

[B28] Mochon D. Schwartz J. Maroba J. Patel D. Ariely D. (2016). Gain without pain: the extended effects of a behavioral health intervention. Manage. Sci. 63, 58–72. 10.1287/mnsc.2015.2322

[B29] Moffitt T. E. Arseneault L. Belsky D. Dickson N. Hancox R. J. Harrington H.. (2011). A gradient of childhood self-control predicts health, wealth, and public safety. Proc. Natl. Acad. Sci. U.S.A. 108, 2693–2698. 10.1073/pnas.1010076108 21262822 PMC3041102

[B30] O'Donoghue T. Rabin M. (2001). Choice and procrastination. Q. J. Econ. 116, 121–160. 10.1162/003355301556365

[B31] Radnitz C. Loeb K. L. DiMatteo J. Keller K. L. Zucker N. Schwartz M. B. (2013). Optimal defaults in the prevention of pediatric obesity: from platform to practice. J. Food Nutr. Disord. 2:1. 10.4172/2324-9323.1000124 25328903 PMC4197992

[B32] Rogers T. Milkman K. L. Volpp K. G. (2014). Commitment devices: using initiatives to change behavior. JAMA 311, 2065–2066. 10.1001/jama.2014.3485 24777472

[B33] Royer H. Stehr M. Sydnor J. (2015). Incentives, commitments, and habit formation in exercise: evidence from a field experiment with workers at a fortune-500 company. Am. Econ. J. Appl. Econ. 7, 51–84. 10.1257/app.20130327

[B34] Samuelson W. Zeckhauser R. (1988). Status quo bias in decision making. J. Risk Uncertain. 1, 7–59. 10.1007/BF00055564

[B35] Schwartz J. Mochon D. Wyper L. Maroba J. Patel D. Ariely D. (2014). Healthier by precommitment. Psychol. Sci. 25, 538–546. 10.1177/0956797613510950 24390824

[B36] Schwartz J. Riis J. Elbel B. Ariely D. (2012). Inviting consumers to downsize fast-food portions significantly reduces calorie consumption. Health Aff. 31, 399–407. 10.1377/hlthaff.2011.0224 22323171

[B37] Steinberg L. Graham S. O'Brien L. Woolard J. Cauffman E. Banich M. (2009). Age differences in future orientation and delay discounting. Child Dev. 80, 28–44. 10.1111/j.1467-8624.2008.01244.x 19236391

[B38] Story M. Resnick M. D. (1986). Adolescents' views on food and nutrition. J. Nutr. Educ. 18, 188–192. 10.1016/S0022-3182(86)80015-2

[B39] Thaler R. H. (2008). Nudge : Improving Decisions About Health, Wealth, and Happiness. New Haven, CT: Yale University Press.

[B40] Wigfield A. Cambria J. (2010). Students' achievement values, goal orientations, and interest: definitions, development, and relations to achievement outcomes. Dev. Rev. 30, 1–35. 10.1016/j.dr.2009.12.001

[B41] Zlatev J. J. Daniels D. P. Kim H. Neale M. A. (2017). Default neglect in attempts at social influence. Proc. Natl. Acad. Sci. U.S.A. 114, 13643–13648. 10.1073/pnas.1712757114 29222183 PMC5748189

