# Supplementary material for: Some Middle School Students Want Behavior Commitment Devices (but Take-Up Does Not Affect Their Behavior)
Source: Front Psychol. 2018 Feb 28;9:206. doi: 10.3389/fpsyg.2018.00206 (PMC5835880; doi:10.3389/fpsyg.2018.00206)
Supplement: Supplementary file 1 [file DataSheet1.pdf]

Supplementary Materials

Some Middle School Students Want Behavior Commitment Devices (But Take-up Does Not Affect Their Behavior)

Carly D. Robinson*, Gonzalo A. Pons, Angela L. Duckworth, Todd Rogers

* Corresponding Author: carlyrobinson@g.harvard.edu

Link to De-identified Data & Analysis Code: https://osf.io/3gm5t/

1 Further Statistics of Factor Analysis for Perceptions about the Paycheck

Supplementary Table 1. Eigenvalues and proportion of variance per factor, without rotation.

Factor	Eigenvalue	Difference	Proportion	Cumulative
Factor1	3.920	2.190	0.392	0.392
Factor2	1.731	0.478	0.173	0.565
Factor3	1.252	0.609	0.125	0.690
Factor4	0.643	0.127	0.064	0.755
Factor5	0.516	0.072	0.052	0.806
Factor6	0.444	0.022	0.044	0.851
Factor7	0.423	0.016	0.042	0.893
Factor8	0.407	0.033	0.041	0.934
Factor9	0.373	0.083	0.037	0.971
Factor10	0.290	.	0.029	1

Supplementary Table 2. Factor loadings and uniqueness of variance, without rotation.

Item	Factor 1	Factor 2	Factor 3	Uniqueness
1	0.613		0.573	0.251
2	0.693		0.420	0.298
3	0.663		0.495	0.282
4	0.670	0.398	-0.428	0.210
5	0.692	0.388	-0.385	0.223
6	0.638	0.415	-0.362	0.290
7	-0.484	0.523		0.482
8	-0.592	0.562		0.331
9	-0.588	0.555		0.347
10	-0.601	0.478		0.383

Note: Scores < 0.3 omitted.

Supplementary Table 3. Eigenvalues and proportion of variance per factor, with Varimax rotation.

Factor	Variance	Difference	Proportion	Cumulative
Factor 1	2.407	0.065	0.241	0.241
Factor 2	2.343	0.189	0.234	0.475
Factor 3	2.153	.	0.215	0.690

Supplementary Table 4. Rotated factor loadings and uniqueness of variance, with Varimax rotation.

Item	Factor 1	Factor 2	Factor 3	Uniqueness
1			0.851	0.251
2			0.778	0.298
3			0.811	0.282
4		0.869		0.210
5		0.851		0.223
6		0.819		0.290
7	0.712			0.482
8	0.798			0.331
9	0.784			0.347
10	0.750			0.383

Note: Scores < 0.3 omitted.

Supplementary Table 5. Factor rotation matrix.

	Factor1	Factor2	Factor3
Factor1	-0.560	0.600	0.571
Factor2	0.816	0.519	0.254
Factor3	0.144	-0.608	0.781

2 Impact of Treatment on Standardized Student Paycheck

Supplementary Table 6. Commitment device and standardized student paycheck results.

Outcome	Standardized Paycheck (1)	Standardized Paycheck (2)	Standardized Paycheck (3)	Standardized Paycheck (4)
Opt-in	0.005 (-0.098, 0.107)	0.005 (-0.110, 0.121)		
Opt-out	-0.022 (-0.123, 0.080)	0.008 (-0.111, 0.128)		
Took Device x Opt-in			0.014 (-0.271, 0.298)	
Took Device x Opt-out			-0.041 (-0.229, 0.146)	-0.039 (-0.240, 0.161)
Analysis	ITT	ITT	TOT	TOT
Excluded		Did not want to set paycheck goal		Control group
N	1,193	900	1,193	788
Coefficients	SD	SD	SD	SD

* *p*<0.1; ** *p*<0.05; *** *p*<0.01

Notes: 95% Confidence Intervals are given in parenthesis. All models control for homeroom, average pre-treatment paycheck, and pre-treatment math grade. “Take-up CD” variables in model 3 and 4 are instrumented using condition assignment (CD = commitment device). Student paychecks are standardized within school and grade.

3 Change in Pre-Post Paycheck by Condition

As a supplement to our main analysis, we do not find evidence that students change in paycheck scores (Post - Pre) differs by condition. Supplementary Table 7 shows a model where we interact treatment conditions with an indicator of post-treatment paycheck. In this table, none of the interaction terms were statistically significant. Additionally, none of the interaction terms were statistically different between each other, $F(2,1192) = 0.49$, $p = .615$. For these analyses we reshaped the dataset, so we had two observations per student: one with the paycheck pre-treatment and a second with paycheck post-treatment. Supplementary Table 8 and Figure 1 show the differences between Post and Pre by condition controlling for covariates. We find no statistically significant changes in paycheck earnings by condition.

Supplementary Table 7. Difference-in-difference analysis on commitment device and student paycheck results.

Outcome	Paycheck
Opt-in	-0.986<br>(-2.866, 0.894)
Opt-out	-0.610<br>(-1.363, 2.583)
Post	-1.684<br>(-3.260, -.108)
Opt-in x Post	0.909<br>(-1.426, 3.245)
Opt-out x Post	-0.244<br>(-2.566, 2.078)
Analysis	ITT
Excluded	
N	2,386
Coefficient	$

* *p*<0.1; ** *p*<0.05; *** *p*<0.01

Notes: 95% Confidence Intervals are given in parenthesis. N represents 1193 students with 2 observations each (pre and post paychecks)

Supplementary Table 8. Difference between Post-treatment Paycheck and Pre-treatment Paycheck by condition

	Post-Pre	95% Confidence Interval
Control	-1.684	(-3.260, -0.108)
Opt-in	-0.775	(-2.499, 0.949)
Opt-out	-1.928	(-3.634, -0.222)

Supplementary Figure 1. Pre-Treatment Paycheck and Post-Treatment Paycheck Trend Line by Condition.

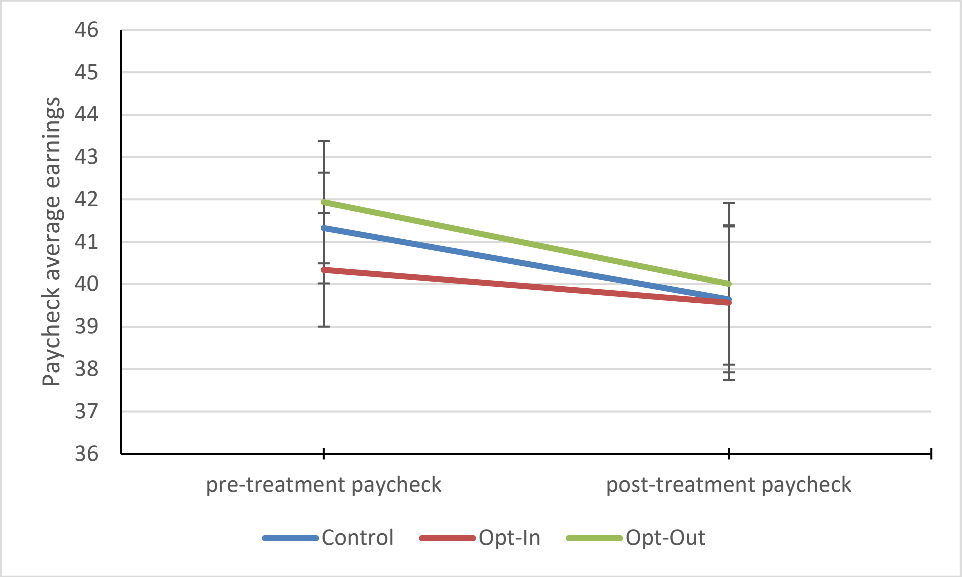

Error bars represent 95% CI

4 Exclusions

Supplementary Table 9. Students Excluded Post-Randomization by Experimental Condition.

	Condition			Total
	Control	Opt-in	Opt-out	
Not Excluded	408	391	406	1,205
	75.56%	72.68%	76.17%	74.80%
Excluded	132	147	127	406
	24.44%	27.32%	23.83%	25.20%
Total	540	538	533	1,611
	100%	100%	100%	100%

χ^2 (2) = 1.983, p = 0.371

5 Adapted Expectancy-Value-Cost (EVC) Scale of student motivation (Kosovich, Hullman, Baron, & Getty, 2015) and practice section

Supplementary Figure 2. Adapted EVC Scale of student motivation.

Page 1

Grade: **Homeroom:**

Part 1.

Directions & Example: There are 10 statements below that you can agree or disagree with. They will be in sentences like this one:

I like ice cream.

You can answer by circling one of the following responses that is most like you.

For ex-

**I like ice cream.**	Strongly Disagree	Disagree	Agree	Strongly Agree

ample, if you like eat ice cream but don't love it, then circle "Agree" like we did here in the example.

Paycheck Statements: For each of the following statements, circle the answer that most represents how you feel.

**1. I know I can earn a better paycheck.**	Strongly Disagree	Disagree	Agree	Strongly Agree
**2. I believe that I can be successful in earning a better paycheck.**	Strongly Disagree	Disagree	Agree	Strongly Agree
**3. I am confident that I can earn a better paycheck.**	Strongly Disagree	Disagree	Agree	Strongly Agree
**4. I think my paycheck is important.**	Strongly Disagree	Disagree	Agree	Strongly Agree
**5. I value my paycheck.**	Strongly Disagree	Disagree	Agree	Strongly Agree
**6. I think my paycheck is useful.**	Strongly Disagree	Disagree	Agree	Strongly Agree
**7. Earning a good paycheck requires too much time.**	Strongly Disagree	Disagree	Agree	Strongly Agree
**8. Because of other things that I do, I don't have time to earn a good paycheck.**	Strongly Disagree	Disagree	Agree	Strongly Agree
**9. I'm unable to put in the time needed to earn a good paycheck.**	Strongly Disagree	Disagree	Agree	Strongly Agree
**10. I have to give up too much to earn a good paycheck.**	Strongly Disagree	Disagree	Agree	Strongly Agree

Turn the Page

[STUDY ID]

Supplementary Figure 3. Practice Page 1.

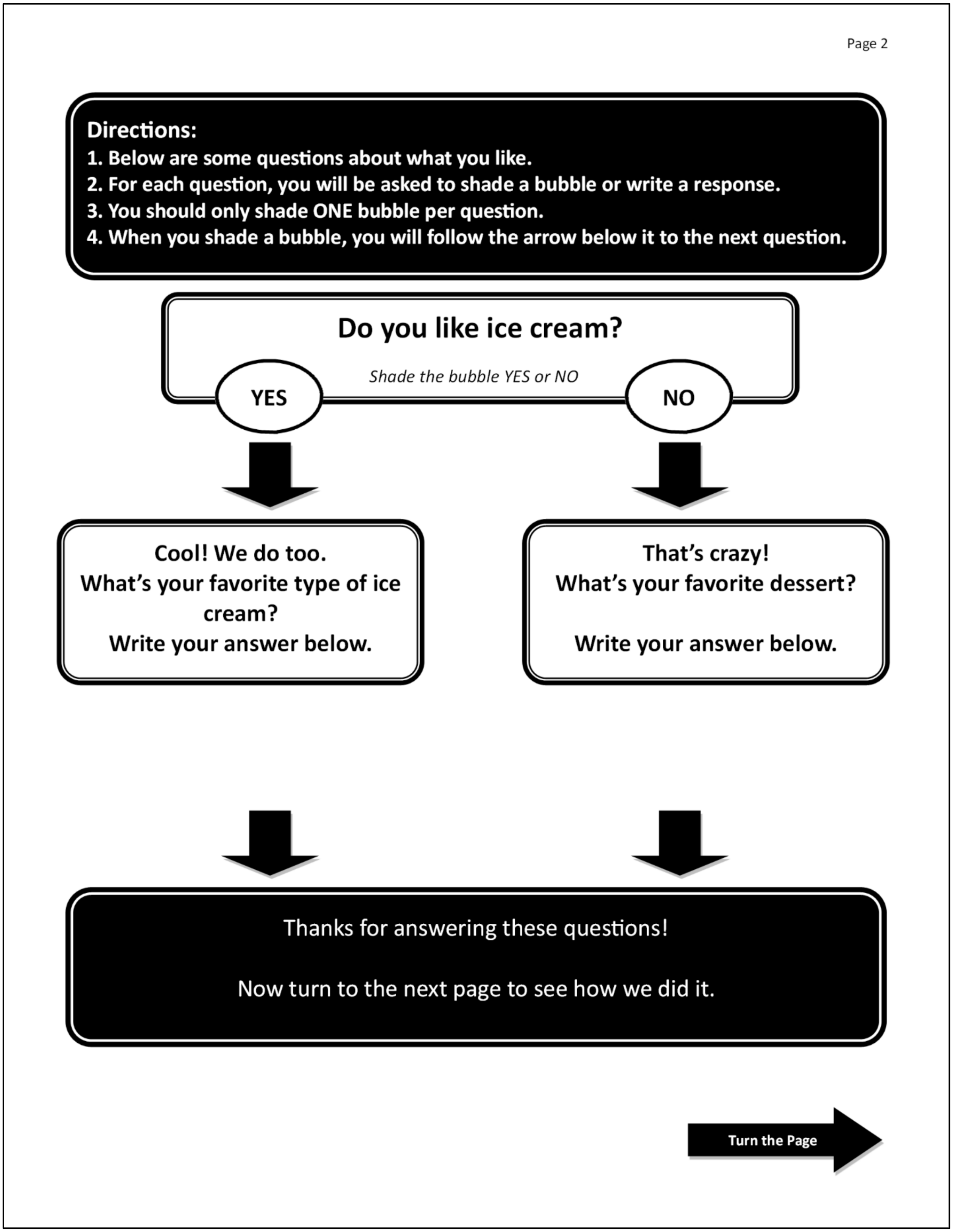

Supplementary Figure 4. Practice Page 2

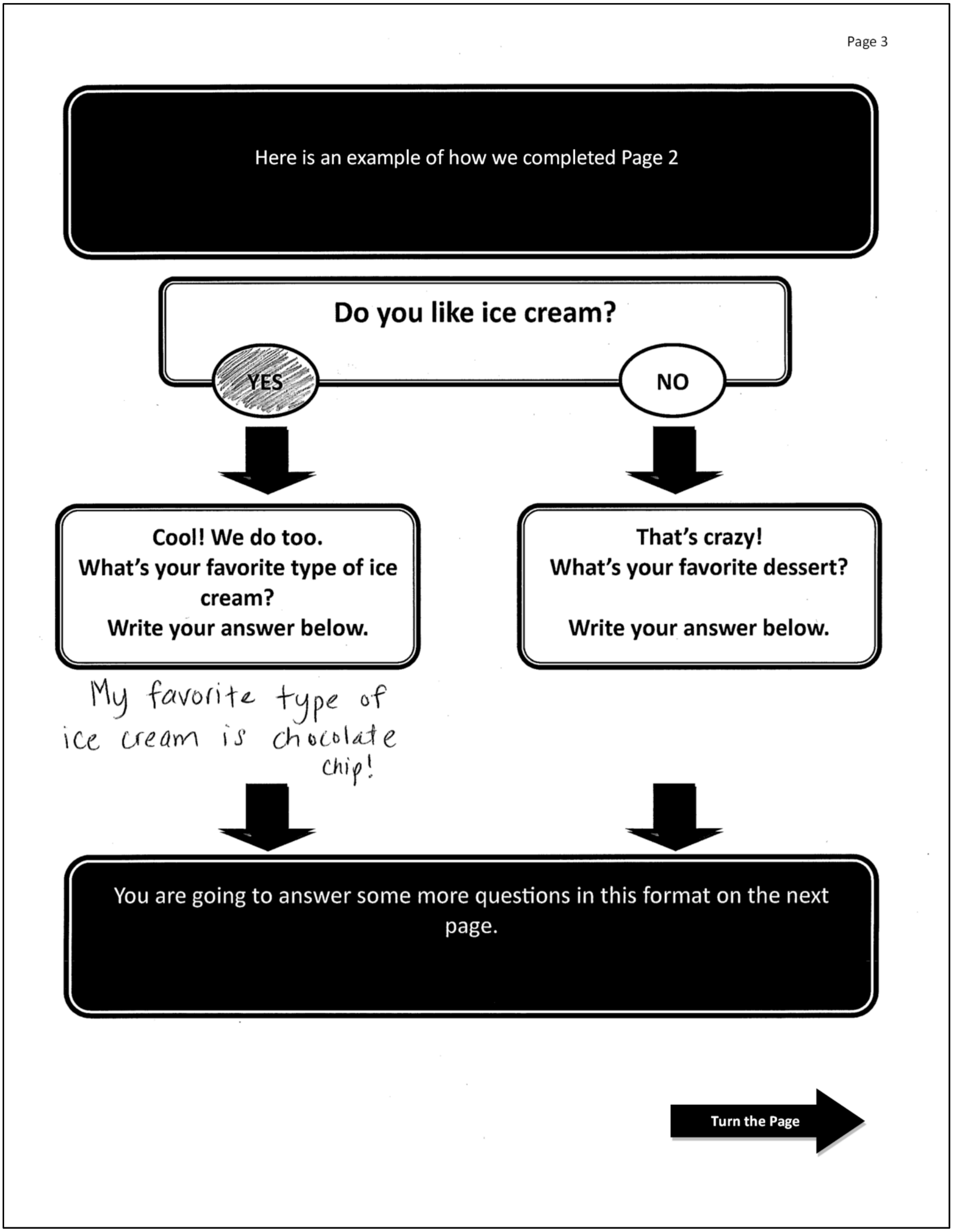

6 Deal/Goal Page

Supplementary Figure 5. Sample Control Deal/Goal Page.

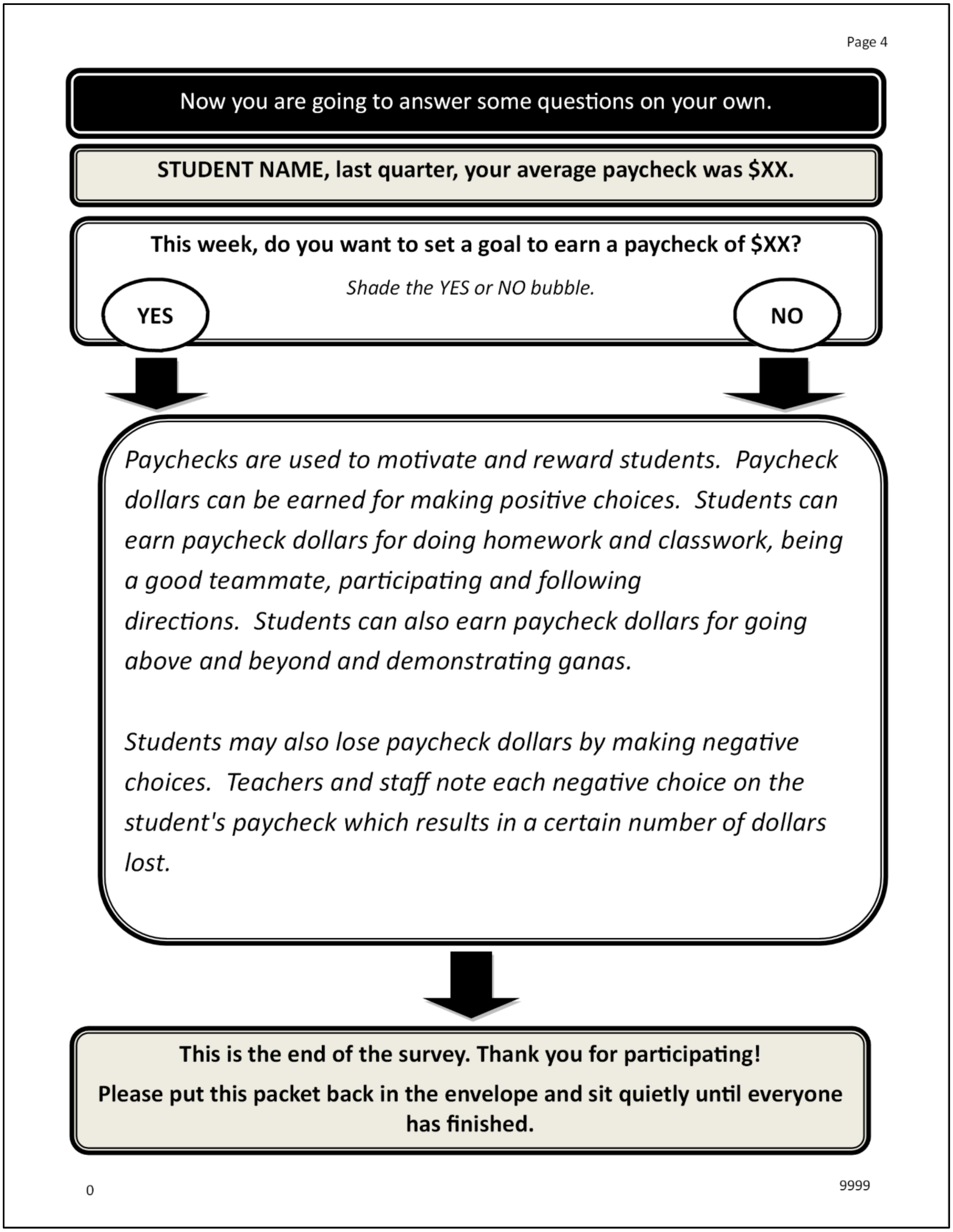

Page 4

Now you are going to answer some questions on your own.

STUDENT NAME, last quarter, your average paycheck was $XX.

This week, do you want to set a goal to earn a paycheck of $XX?

Shade the YES or NO bubble.

YES **NO**

Paychecks are used to motivate and reward students. Paycheck dollars can be earned for making positive choices. Students can earn paycheck dollars for doing homework and classwork, being a good teammate, participating and following directions. Students can also earn paycheck dollars for going above and beyond and demonstrating ganas.

Students may also lose paycheck dollars by making negative choices. Teachers and staff note each negative choice on the student's paycheck which results in a certain number of dollars lost.

This is the end of the survey. Thank you for participating!

Please put this packet back in the envelope and sit quietly until everyone has finished.

0 9999

Supplementary Figure 6. Sample Opt-in Deal/Goal Page.

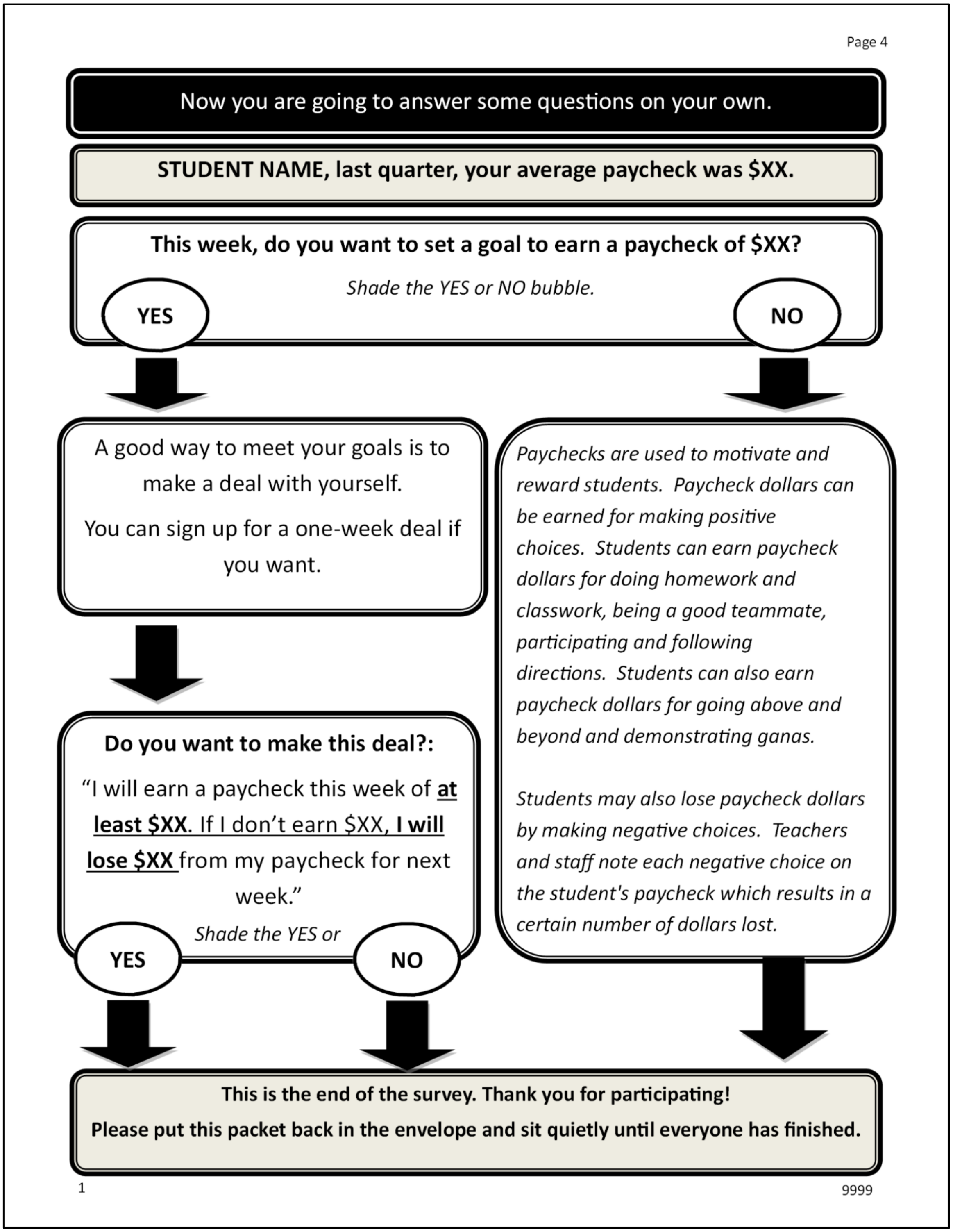

Page 4

Now you are going to answer some questions on your own.

STUDENT NAME, last quarter, your average paycheck was $XX.

This week, do you want to set a goal to earn a paycheck of $XX?

Shade the YES or NO bubble.

YES **NO**

A good way to meet your goals is to make a deal with yourself.

You can sign up for a one-week deal if you want.

Do you want to make this deal?:

"I will earn a paycheck this week of **at least $XX**. If I don't earn $XX, **I will lose $XX** from my paycheck for next week."

Shade the YES or

YES **NO**

Paychecks are used to motivate and reward students. Paycheck dollars can be earned for making positive choices. Students can earn paycheck dollars for doing homework and classwork, being a good teammate, participating and following directions. Students can also earn paycheck dollars for going above and beyond and demonstrating ganas.

Students may also lose paycheck dollars by making negative choices. Teachers and staff note each negative choice on the student's paycheck which results in a certain number of dollars lost.

This is the end of the survey. Thank you for participating!

Please put this packet back in the envelope and sit quietly until everyone has finished.

1 9999

Supplementary Figure 7. Sample Opt-out Deal/Goal Page.

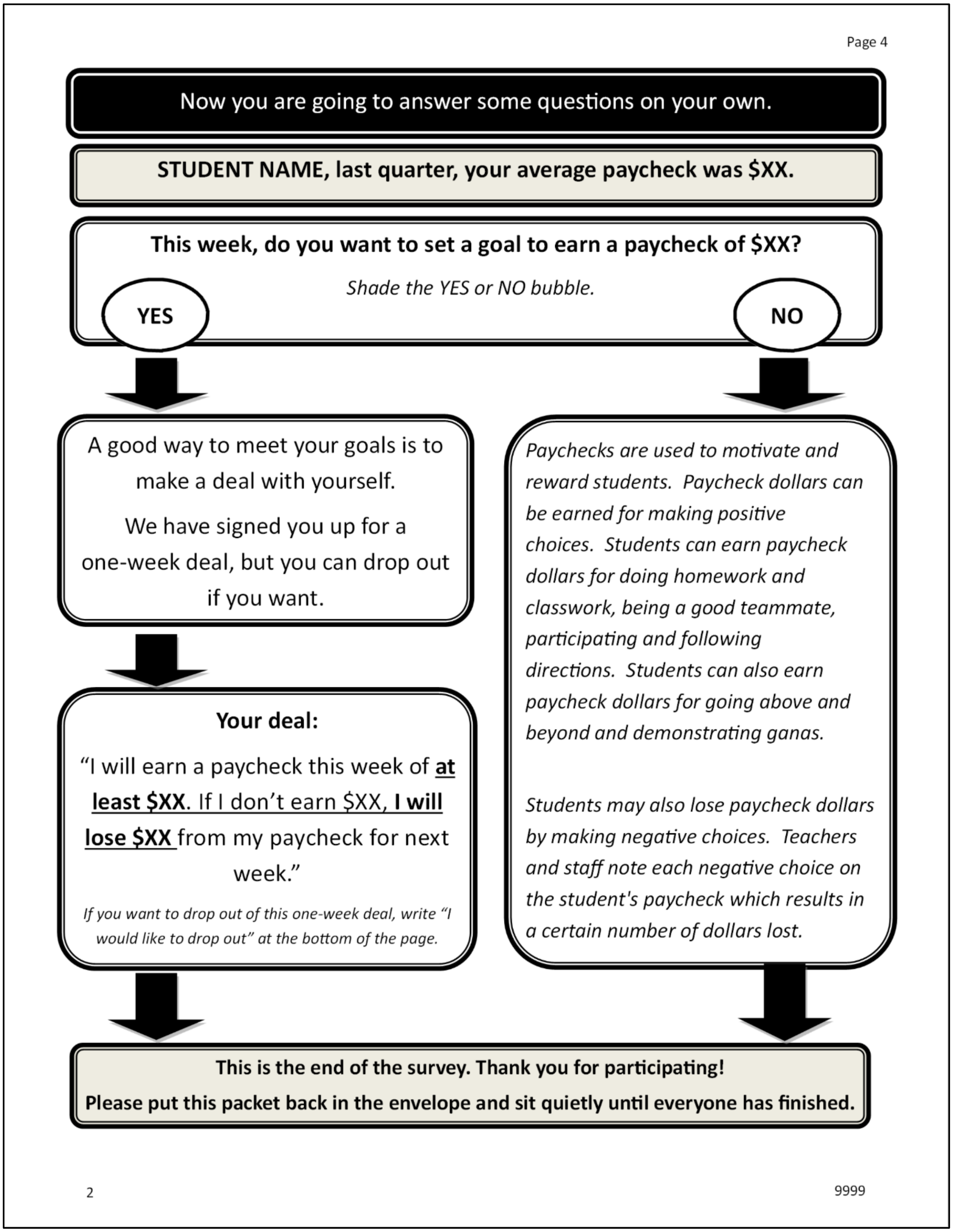

Page 4

Now you are going to answer some questions on your own.

STUDENT NAME, last quarter, your average paycheck was $XX.

This week, do you want to set a goal to earn a paycheck of $XX?

Shade the YES or NO bubble.

YES **NO**

A good way to meet your goals is to make a deal with yourself.

We have signed you up for a one-week deal, but you can drop out if you want.

Your deal:

"I will earn a paycheck this week of **at least $XX**. If I don't earn $XX, **I will lose $XX** from my paycheck for next week."

If you want to drop out of this one-week deal, write "I would like to drop out" at the bottom of the page.

Paychecks are used to motivate and reward students. Paycheck dollars can be earned for making positive choices. Students can earn paycheck dollars for doing homework and classwork, being a good teammate, participating and following directions. Students can also earn paycheck dollars for going above and beyond and demonstrating ganas.

Students may also lose paycheck dollars by making negative choices. Teachers and staff note each negative choice on the student's paycheck which results in a certain number of dollars lost.

This is the end of the survey. Thank you for participating!

Please put this packet back in the envelope and sit quietly until everyone has finished.

2 9999

7 Reminder notices

Supplementary Figure 8. Reminder Control + Declined Goal.

Student Engagement at [SCHOOL] Study

Dear [STUDENT FIRST NAME],

Thank you for taking the survey yesterday!

From:

- The Harvard University Research Team

[STUDENT FULL NAME] GRADE:
HOMEROOM:

Supplementary Figure 9. Reminder Took Goal – Declined Commitment Device.

Student Engagement at [SCHOOL] Study

Dear [STUDENT FIRST NAME],

Thank you for taking the survey yesterday!

Reminder: You hope to earn $GOAL on your paycheck this week.

From:

- The Harvard University Research Team

[STUDENT FULL NAME]
*

GRADE:
HOMEROOM:

Supplementary Figure 10. Reminder Took Commitment Device.

Student Engagement at [SCHOOL] Study

Dear [STUDENT FIRST NAME],

Thank you for taking the survey yesterday!

Reminder: You took the deal on the survey. You chose to earn $GOAL on your paycheck this week. If you don't earn $GOAL, you will lose $DEDUCTION on your next paycheck.

From:

- The Harvard University Research Team

[STUDENT FULL NAME] GRADE:
** HOMEROOM:

8 Notification post treatment

Supplementary Figure 11. Post Treatment Notification Generic.

Student Engagement at [SCHOOL] Study

Dear [STUDENT FIRST NAME],

Thanks again for participating in the research activity. We hope you are proud of your paycheck and effort this week.

From:

- The Harvard University Research Team

[STUDENT FULL NAME]

GRADE
HOMEROOM

Supplementary Figure 12. Students who took deal and did not meet goal.

Student Engagement at [SCHOOL] Study

Dear [STUDENT FIRST NAME],

You chose to take the deal on the survey last week. You agreed to earn $GOAL on your paycheck this week. If you did not earn $GOAL, you would lose $DEDUCTION on your paycheck next week.

Unfortunately, you did not earn $GOAL, so $DEDUCTION will be taken from your paycheck next week.

From:

- The Harvard University Research Team

[STUDENT FULL NAME] GRADE
HOMEROOM

Supplementary Figure 13. Students who took goal and met goal.

Student Engagement at [SCHOOL] Study

Dear [STUDENT FIRST NAME],

Way to go! You met your goal of earning $GOAL on your paycheck this week. Your hard work and persistence paid off. We hope you are proud of your paycheck and effort.

Congratulations!

From:

- The Harvard University Research Team

[STUDENT FULL NAME]

GRADE

HOMEROOM
